# Fighting Pressures: Successful Psychosocial Adjustment of Middle Eastern Students at US Universities

**DOI:** 10.3389/fpsyg.2021.747321

**Published:** 2021-10-05

**Authors:** Waleed B. Al Abiky

**Affiliations:** Department of Curriculum and Instruction, College of Education, Qassim University, Buraidah, Saudi Arabia

**Keywords:** successful adjustment, psychosocial, Middle Eastern students, US universities, pressure

## Abstract

Middle Eastern students seeking academic degrees in the US universities often experienced array of challenges in multiple levels as they adjust to the new environments. The current study investigated some of the psychosocial factors affecting their adjustments, namely: age, gender, and marital status. Quantitative method was used to collect data in which a survey was distributed and later analyzed for 92 Middle Eastern participants. The results of the study reveal the followings: (1) age was a significant factor impacting the level of adjustment at α = 0.0001, (2) gender on the other hand was not statistically significant as there was no difference between males versus females on the level of adjustment, (3) results also revealed that marital status had a statistically significant impact in which married participants displayed a higher level of adjustment than singles, and (4) with the current changes in the social lives and gender roles in the Middle East, further investigations are opened for exploration.

## Introduction

Feeling insecure in a new country is just a prevalent burden and a challenging transition to make (Cely-Santos and Hernandez-Manrique, 2021). In particular, Middle Eastern immigrants have doubled in the last 5 years from 25 to 54 million, according to Pew Research Centre analysis of data from United Nations agencies. Moreover, the main cause for the surge in immigrants’ number was due to the armed conflict and forced displacement for millions of people. The United Nation data reported that the Middle Eastern people are the fastest growing number of migrants due to the forcibly displacement (Pew Research Centre, 2020).

In the United States alone, the number of Middle Eastern population immigrated there is nearly reaching 3.5 million (U.S. Census Bureau, 2021). Those Middle Eastern reside in all the 50 states including Washington, D.C., and 94% reside in the metropolitan area of the some major cities (Arab American Institute, 2021). Seeking educational degrees has often been one way to survive and cope with the social and environmental changes.

Across US colleges and universities, the number of foreign students studying there has lately significantly increased. According to Open Doors (2019), the annual report on international academic mobility published by the Institute of International Education (IIE), there were more than 1,075,496 international students studying in the United States in the academic year 2019/2020. In the United States, the total number of Middle Eastern students enrolled in the US colleges and universities in the academic year 2019/2020 reached 72,325, which constitutes approximately 6% of the overall number of international students studying in the US colleges and universities.

However, recent studies showed that foreign students are a clear sample at high risks of developing some psychological issues and problems due to the heavy pressure put on their shoulders as a result of their new social and academic environments such as anxiety, homesickness, and poor mental and emotional health (Pan et al., 2008; Yan and Berliner, 2013). Indeed, many or maybe all international students experience a whole range of psychosocial adjustment difficulties, including homesickness, language barriers, isolation, and loneliness. Yet, the factors that might ease the heavy burden and transition process remain yet unexplored.

## Literature Review

### Successful Adjustment

Adjustment has a Latin root, *ad jure* that means “to bring or make right.” Our lives are about a continuous change and overcoming challenges in the environment to make life right or feel right. Berry (1979) also defined adjustment as the degree to which one feels harmony and in congruent with the environment. However, integrating into a host social and academic life of a university, a city, and a county is indeed a challenging process.

The psychological adjustment has been defined as the psychological process and product that includes handling of new social values, food, weather, standards, and norms for individual successful acceptance and indulgence (Jain, 2012; Gündüz and Alakbarov, 2019). This generates many enduring challenges for international students. Adjustment for international students means the good internal and external balance between continuity and adaptation, and between self and others.

### Psychological Harms

Recent research findings show that international students in general are a population at high risk of having some psychological problems or suffering from poor mental and psychological health due to array of difficulties such as stress and anxiety (Pederson, 1991; Pan et al., 2008). International students are different from any other group of non-resident visitors because of their academic goals, the desire to do well academically and even socially adds another level of anxiety and complexity for students’ social and cultural adjustment (Ward et al., 2001; Darwish, 2015).

However, although an extensive research has been conducted to understand or identify the factors harming or hindering the international students’ process of adjustment, the current literature has not yet examined or even sufficiently listed the factors that help international students to cope with these difficulties and ease the adjustment process (Brunsting et al., 2018). Current studies found some significant factors that hinder or even affect the process of adjustment such as language barriers, cultural shock, and the separation from family support and accustomed lifestyle in the home country (Pederson, 1991; Kempt, 1999; Standford Report, 2004; Rubin, 2017), Little is known, if any, about the factors helping those international students from Middle East in their adjustment process.

### Predictors

Broadly speaking, most recent research indicated that international students face an array of challenges and difficulties adjusting to the new educational settings (Rivas et al., 2019). However, it has been found that younger students have more difficulties than older ones in making the adjustment. Konyu-Fogel (1993) pointed out that older international students reported significantly less difficulties in academic adjustment than younger ones. Shabeeb (1996) determined that younger Saudi and Arabian Gulf students usually face more problems than older ones. However, age alone might not be a very precise predictor of adjustment; it might work with other factors.

In socio-cultural studies, there is a continuing discussion about gender’s effects on sociocultural adjustment. Female students, for instance, seem to face more adjustment problems and difficulties than males. Emotional, psychological, and self-perception are some of the difficulties faced by females, while male students experience more difficulties in English competence (Konyu-Fogel, 1993).

Rajapaksa and Dundes (2002-2003) found international female students to be less successful in adjustment than men. Homesickness, for instance, was found to be more prevalent with women at 36% compared to 22% of men. Also, females felt to be less likely satisfied with their social network and less likely to be engaged even in a romantic relationship with another on the same campus.

In seeing language proficiency as a significant factor affecting the process of adjustment of international students at American Universities positively or negatively, some researchers found that two significant uncertainties faced by international students were their competency of the English language and making American friends. A study done by Kempt (1999), moreover, shows significant relationships between anxiety and depression related to the English proficiency of international students. Low English proficiency tends to limit students’ social interactions with members of their own culture. Because of their limited English skills, they have difficulties in accessing resources that can assist them in their adjustment.

Researchers agree that the English proficiency level is crucial to successful adjustment. English language for many international students is usually a big hurdle. Shabeeb (1996) found that the most problematic area for Saudi and Arabian Gulf students was the English language. Researchers suggested that a higher English proficiency level contributes positively with adjustment. Good English, of course, enables international students to make better progress not only in academic studies but also in their social lives as well. Low proficiency level, on contrast, often hinders the process of adjustment.

As for the significant impacts of marital status, research findings were mixed. Some research addressed the importance of the availability of a strong support person to whom international students can turn to in crises. Some researchers believed that having supportive family members, faculty, and/or classmates was instrumental in students’ adjustment and acceptance to college campuses. The findings of some researchers indicate that marital status has no significant impacts on adjustment of Middle Eastern students (Shabeeb, 1996). On the other hand, other researchers showed that marital status has a significant influence on adjustment. Researchers, however, do not often indicate or even state whether marital status exerts positive or negative impacts on adjustment. Yet, it, students’ marital status, has, of course, a great influence on their adjustment.

Furthermore, although the majority of cross-cultural studies have often focused merely on the challenges and difficulties often encountered by the international students, some research has also found that international students have good academic achievements, positive attitudes toward their educational institutions, and recognizable success in their adjustments. It is more important and reasonable to look at the strengths and success of those international students, and Middle Eastern are not exception, in adjusting themselves to a new culture, food, weather, and educational system. However, as the saying goes “behind every success, there is a genuine helper,” this study investigated the impact of the following psychosocial factors: age, gender, and marital status of Middle Eastern students studying in the United States.

The current study aimed to statistically investigate the significance of some factors relating to the psychosocial successful adjustment of Middle Eastern students at the US universities. The main question of the current study can be stated as follows:

I. To what extent subjects’ age, gender, and the marital status be related to the successful psychosocial adjustment of Middle Eastern students?

## Materials and Methods

To achieve the goal of the study, quantitative method was used in which a survey was distributed to the participants. Subjects’ responses were collected and statistically analyzed. All participants were Middle Eastern students studying in the US universities who were only on students’ visa and seeking an academic degree.

### Design

In the current study, analytical cross-sectional was the design of the study. Subjects were divided into groups for each independent variable. Each participant was assigned into only one group. For instance, in regard to the age as an independent variable, each subject was assigned to one group:

➢ Group 1: subjects under 25 years of age.
➢ Group 2: subjects between 26 and 35 years of age.
➢ Group 3: subjects above 35.

The criterion variable in the study is “the level of successful psychosocial adjustment.” Subjects were asked in the questionnaire to provide ratings in their responses to what extent he/she agrees or disagrees with each statement. There were a total of 16 statements. Subjects were prompted to respond to each item by circling a number between 1 and 5. The possible scores on the scale may range from a low of “16” (if a subject circled “1” for each item) to a high of “80” (if a subject circled “5” for each item). The higher the score is, the more successful the participant is in the process of adjustment.

To assess the criterion variable (level of successful psychosocial adjustment), the possible scores were divided into five categories or levels. Each level indicates the strength of adjustment.

1. Scores ≥ 41 indicate weak adjustment.
2. Scores from 42 to 54 indicate moderate adjustment.
3. Scores from 55 to 67 indicate successful adjustment.
4. Scores ≤ 68–80 indicate perfect adjustment.

Therefore, the sum of the scores for each group would indicate their level of adjustment. One of the major sources of this categorization was the Acculturative Stress Scale for International Students (ASSIS) (Sandhu and Asrabadi, 1994, 1998). In their study, Sandhu and Asrabadi assessed international students’ perceptions of stresses experiences with a total sample of 128. The possible scores ranged from 36 to 180, with higher scores indicating higher levels of adjustment.

### Participants

Participants were males and females, graduates and undergraduates, Middle Eastern students enrolled in the fall of 2018 at one of the US universities. The participants in the study were selected randomly regardless of their field of study. Moreover, the Ethical Treatment of Subjects as stated in APA was adhered to in the current study in which the rights and interests of human subjects were protected. Participants who fit the criteria were invited, and consent forms were signed. One-hundred and eight (108) questionnaires were distributed, and 93 were returned. One survey was excluded due to the missing data. In addition, the study excluded participants who were: (1) immigrant students originated from the Middle East, (2) workers or non-degree students, or (3) students under 17 years old.

The final sample consisted of 92 participants, Middle Eastern students pursuing degrees in US universities, of which 61 (66%) were males and 31 (34%) were females. Of the sample, 60 (65%) were graduate and 32 (35%) were undergraduate. Singles were 34 (37%) and married 58 (63%) of the total sample.

### Apparatus

The instrument obtained specific information regarding age, gender, and marital status. The questionnaire also requested responses to 16 statements on a five-point Likert-type scale, and subjects responded to each statement by selecting a number from “1” to “5” in which *1 = Strongly disagree, 2 = Disagree, 3 = No opinion, 4 = Agree*, and *5 = Strongly agree*. As a result, the possible scores on the attitude scale ranged from a low of “20” (if a subject selected number “1” for each item) to a high of “100” (if a subject selected “5” for each item).

These statements focused mainly on some areas that likely have impacts on students’ adjustment such as academic coursework, social relationship, developing friendships, and maintaining cultural customs and practices such as “I am fully engaged with my classes” and “I feel comfortable here.”

Upon getting the approval from the Institutional Review Board (IRB) at the University of Arkansas, Middle Eastern students were reached. Other Middle Eastern students were also asked to distribute the questionnaire to other Middle Eastern students. Consent was sought verbally and in written form and attached to the survey. The participants, all of them, were prompted not to put their names, or indicate any identification information on the research instrument. The questionnaire was completely anonymous and used for research purposes only. There were no risks, no credits, or gifts for participation.

## Results

### Age as a Predictor

Age of subjects was the first independent variable. Each subject was assigned to a chronological age category. The data were arranged into age range categories. Table 1 illustrates the distribution of participants regarding the predictor variable “age.”

**TABLE 1:** Frequencies and percentages of age groups for the 92 participant.

**Age group**	***N***	**Percent**	**Cumulative percent**	**Mean**	**SD**
≥20	26	28		49.038	9.56
20–30	44	47.83	76.09	57.63	7.56
≤30	22	23.91	100.00	60.227	5.66
Total	92	100			

As shown in Table 1, 28% of the sample were less than 20 years (26 subjects), 48% were between the age of 20 and 30 (44 subjects), and 24% were above 30 (22 subjects).

On the criterion variable (level of adjustment), the mean score for group 1, subjects’ under 20 years old, was 49.038 (SD = 9.56), the mean for group 2, those subjects between 20 and 30 years old, was 57.636 (SD = 7.56), and the mean for group 3, subjects above 30 years old, was 60.227 (SD = 5.65). Subjects in the first group have a moderate level of adjustment, while subjects in groups 2 and 3 have successful adjustment. The findings are just an indicator that age is a significant factor for the adjustment of the Middle Eastern students. As subjects get older, the more adjusted they become.

One-way ANOVA was then used to test the impact of subjects’ age on their adjustment. Having interring the data to be analyzed by SAS (Hatcher, 2003), the following were used:

• The predictor variable was “subjects’ age.” This was a limited-value variable because subjects selected only one of the three values (1 = less than 20, 2 = between 20 and 30, and 3 = above 30). This predictor variable was assessed on a nominal scale. Each subject is exposed to just one condition or group under the predictor variable.
• The criterion variable was “level of adjustment.” This was a numerical variable, and it was assessed on a ratio scale because it has equal intervals and a true zero point.

Results of analysis using a one-way ANOVA with one between-subjects factor are summarized in Table 2.

**TABLE 2:** ANOVA summary investigating the relationship between age and level of adjustment.

**Source**	**df**	**SS**	**MS**	***F***	***p***	***R* ^2^**
Subjects’ age	2	1768.21	884.10	14.53	<0.0001	0.25
Within groups	89	5415.01	60.84			
Total	91	7183.22				

*N = 92.*

The analysis revealed a significant treatment effect for the level of adjustment, *F*(2,89) = 14.53, *MSE* = 60.84, *p* < 0.0001. This means that subjects’ age (the independent variable) had statistically significant impacts on the adjustment of Middle Eastern students at the US universities. In the analysis, *R*^2^ was computed as 0.25. This is the effect size indicating that the subjects’ age accounted for 25% of the variance in the subjects’ level of adjustment.

The findings also provide a support for the research’s hypothesis that (a) subjects who are under 20 years of age will demonstrate a lower level of adjustment than subjects who are above 20 years of age and (b) the older the subjects get, the more successfully adjusted they become. However, the findings failed to provide the support for the hypothesis that subjects who are between 20 and 30 will demonstrate a statistically significant lower level of adjustment than subjects who are above 30 years old.

Moreover, Tukey’s HSD test showed that subjects in group 2 and group 3 scored significantly higher on adjustment than did subjects in group 1, subjects under 20 years old (*p* < 0.05). With α set at 0.05, there were no significant differences between subjects in group 2 and subjects in group 3. Table 3 presents the confidence intervals for differences between the means.

**TABLE 3:** Results of Tukey test comparing (A) group 1, subjects under 20 years old, (B) group 2, subjects between 20 and 30, and (C) group 3, subjects above 30 years old, on the criterion variable (level of adjustment).

**Comparison^a^**	**Difference between means**	**Simultaneous 95% confidence limits**	
		**Lower**	**Upper**
C–B	2.591	−2.264	7.446
C–A	11.189*	5.803	16.575
B–A	8.598*	3.999	13.197

*N = 92.*

*^a^Differences are computed by subtracting the mean for the second group from the mean for the first group.*

*Tukey test indicates that the difference between the means is significant at p < 0.05.

### Gender as a Factor

Male participants were more than females. Of the total sample, (66%) were males, and (34%) were females. This was expected because females, in the Middle Eastern culture, have more restricted roles than males. As a result, males who study abroad usually outnumbered females.

Gender as an independent variable was tested using a comparison of means. Independent-samples *t*-test was used. Table 4 shows the results summary for investigating the mean scores.

**TABLE 4:** *t*-Test summary table investigating the mean scores difference between male versus female subjects.

**Subjects**	***N***	***M***	**SD**	***T***	***p***	***d***	***F***	**Pr > F**
Male	61	56.393	9.0116	−0.86	0.3932	0.19	1.08	0.8334
Female	31	54.71	8.6648					

Results were analyzed using an independent-samples *t*-test. This analysis revealed a non-significant difference between the two groups, *t*(90) = −0.86, *p* = 0.3932. The findings fail to provide support for the study’s research hypothesis. These findings also fail to reject the null hypothesis.

The sample means show that male subjects displayed a mean score on adjustment similar to that displayed by female subjects (for males, *M* = 56.393, SD = 9.0116; for females, *M* = 54.71, SD = 8.6648). The observed difference between the means was −1.684, and the 95% confidence interval for the difference between means extended from −5.583 to 2.2152. The effect size was computed as *d* = 0.19. According to Cohen’s (1969) guidelines, this represents a relatively small effect, which means that there is no big or significant difference between males and females.

As Table 4 shows, the results also failed to reject the null hypothesis. From the “Equality of Variances” produced by SAS, PROC TTEST also computed an “F” statistic to test the null hypothesis, that in the population, there is no difference between male subjects versus females on the criterion variable (successful adjustment) with respect to their variances. Since the *p*-value for the resulting “F” test is Pr > F 0.8334, which is greater than 0.05, the null hypothesis is accepted and concludes that the variances are equal.

As shown from *t*-test output, gender has no significant impacts on adjustment of Middle Eastern students at US universities. Males and females have a similar level of adjustment and, may be, go through the same adjustment process. The differences between the two groups in their adjustment can be attributed to other variables such as their personal characteristics, but not to their gender.

**FIGURE 1:**
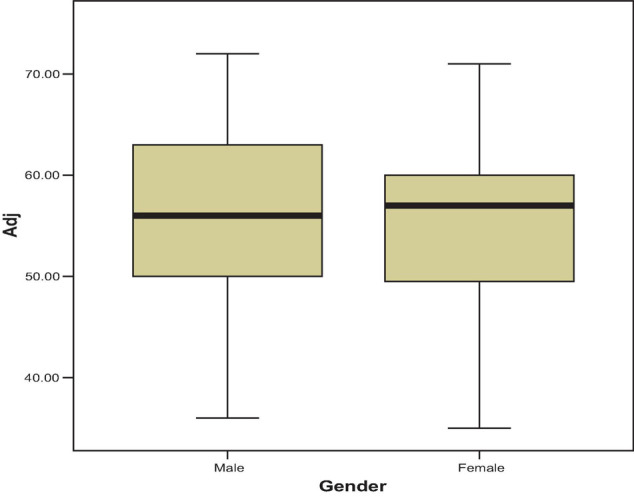
Box plots of adjustment level for male and female.

In addition, Figure 1 is a box-and-whisker plot illustrating the comparison between the two groups. The box depicts the distribution of adjustment’s scores for males and females of Middle Eastern students.

Studying the box plot, I can conclude that females received scores similar or close to the scores of males. The distribution of scores for female Middle Eastern students is highly negatively skewed because the distance from the median to Q1 is much greater than the distance from median to Q3. The variability of scores is much greater in female scores. There are no outliers in both groups.

### Obtained Statistic for Marital Status

The survey contains four options for the marital status section, which are single, married, divorced, and other. None of the participants checked divorced or other items. As a result, these two options were eliminated and changed into dichotomous: singles group and married group. Of the total sample, 37% of the total subjects were singles and 63% were married. To examine the impacts of marital status of Middle Eastern students, *t*-test was used and run (Table 5).

**TABLE 5:** *t*-Test summary table for study investigation the mean scores difference between single subjects versus married subjects.

**Subjects**	***N***	**Percent**	***M***	**SD**	***T***	***p***	***F***	**Pr > F**
Single	34	36.96	51	9.4163	−4.37	<0.0001	1.68	0.0831
Married	58	63.04	58.655	7.2561				

*d = 0.94.*

Results were analyzed using an independent-samples *t*-test. This analysis revealed a significant difference between the two groups, *t*(90) = −4.37, *p* < 0.0001. These findings provide support for the study’s research hypothesis; that’s married subjects will display a higher mean score than female subjects on the level of adjustment. These findings also provide support to reject the null hypothesis. The sample means show that the married subjects scored significantly higher on adjustment compared to single subjects (for married subjects, *M* = 58.655, SD = 7.2561; and for single subjects, *M* = 51, SD = 9.4163). The observed difference between the means was −7.655, and the 95% confidence interval for the difference between means extended from −11.14 to −4.173. The effect size was computed as *d* = 0.94. According to Cohen’s (1969) guidelines, this represents a relatively large effect.

Comparing the means of the two groups, single subjects have moderate adjustment, while married subjects have strong adjustment. I can conclude that marital status has significant impacts on Middle Eastern students at the US universities. Being married is an important factor in helping Middle Eastern students to successfully adjust to their academic and social lives alike.

## Discussion

The study here aimed to statistically examine the impacts of some factors, which are age, gender, and marital status on the level of adjustment of Middle Eastern students at some of the US universities. The main issue here is to find out the degree to which each predictor variable is effective on the criterion variable. Specifically, the findings suggest that some of these factors, namely, age and marital status, are significant factors which can greatly affect the process of adjustment of Middle Eastern students at these US universities. It appeared that marriage might make the process of adjustment smoother and easier for Middle Eastern students. On the other hand, the findings also indicate that gender was not a significant factor in itself in the process of adjustment. Males and females have the same chances and challenges in the process of adjustment.

The results here also indicate that none of the groups reached the perfect level of adjustment to the academic and social lives. There were indeed some individuals who had statistically perfect adjustment, but as groups, none of these groups was perfectly adjusted. On the other hand, none of these groups failed to adjust or even had a very weak adjustment. It was found that some individuals failed to adjust or have a very weak level of adjustment.

The results in this study show that subjects’ age has significant impacts on the adjustment of Middle Eastern students. As subjects get older, the more adjusted they become. This finding was consistent with some of the previous research findings, which reveal that younger students have more difficulties in making adjustments. Shabeeb (1996) found that younger Saudi and Arabian Gulf students encounter more problems than older ones. Obviously, younger and older students have advantages and disadvantages in making adjustments. Yet, compared with older students, younger Middle Eastern students are probably less mature and have less experience, which may cause more difficulties for them in their new environment. Older students, especially graduate, might be more mature in dealing with problems, and might be more trained and prepared in their fields of study.

However, age alone and in itself may not be a very precise and sufficient factor to predict their adjustment. It might be a wise thing to do to combine age with other factors that might have great impacts on adjustment such as social support and friendship to predict adjustment.

As for gender’s impact on adjustment, obtained results revealed that there was no difference between males and females in the process of adjustment. They, males and females, encounter the same range of difficulties and challenges. They both accomplish some success and become perfectly adjusted to the new situation. These difficulties and success in adjustment can be attributed and related to some other factors such as personal characteristics, not to their gender.

These findings were a bit surprising, because females in general might have greater needs for connection with others and be more demanding for personal harmony and supports than males. As in almost every society, men in the Middle East have dominant gender’s roles. They, men in Middle Eastern culture, have a predominant position in public importance and appearance. Yet, it seems in this study that in the process of successful adjustment, gender’s roles did not lead subjects to negative outcomes. As shown in my results, males and females have equal opportunities and challenges in the process of adjustment.

Regarding gender’s impacts on subjects’ adjustment, the findings here were not perfectly consistent with previous studies and research. Studies, in general, seem to conclude that female international students experience more adjustment problems than male students. Konyu-Fogel (1993) found that female international students experienced significantly more academic adjustment difficulties than male subjects. Shabeeb (1996) found that Saudi and Arabian Gulf female students faced more problems in the area of academic and social adjustment.

Additionally, the results did show that there were significant differences between married Middle Eastern students and single ones. It seems that married Middle Eastern students were more successful in their adjustment than singles. This can be explained by findings that consider positive social support as a mitigating factor in adjustment. These findings were not also consistent with some previous studies.

Shabeeb (1996) noted that there were no significant differences among married and single students from the Arabian Gulf students in adjustment difficulties and concerns. It can be said that marriage gives Middle Eastern students confidence and support and provides someone to turn to in crises.

There is clearly a need for continued research in this area using quantitative and qualitative methodologies. A major problem faced by the researcher here has been the lack of research and studies about Middle Eastern students’ adjustment, in particular. Few, if there is any, studies widely examined some factors contributing to the successful adjustment of Middle Eastern students. However, there were a plenty of research about International students as a whole examining a whole range of difficulties and challenges faced by international students.

Furthermore, variables that are more relevant can also be explored and investigated. Variables such as income and length of stay are greatly relevant, and can have great impacts on the adjustment of Middle Eastern students. It is also possible and beneficial to examine the factors that might cause a failing or weak adjustment among Middle Eastern students. Diagnosing closely the problems, we might be able to find effective remedies.

## Limitations and Future Directions

Although the present research has some strengths such as filling the gap in the current literature as there have been few studies which investigated the topic with the same variables on Middle Eastern students, there are some limitations. One of the limitations is that it is based on university students seeking educational degrees in the United States. Future research could and should expand the sample to include non-university students and other cultural groups other than Middle East.

Another limitation is that the study was conducted in 2018 targeting Middle Eastern students in US universities. Many things have happened, and future research might focus on cultural impact on subjects’ responses to real-life situations and events. Moreover, personality types might also be a focus as in relation to adjustment as there is a clear relevance to personality types and adjustment with the environments.

## Conclusion

It seems that if Middle Eastern students can have better language ability prior to their coming to the United States, be more open to American societies, and have a companion, they would have a better and faster adjustment. Since there is a mutual relationship between Middle Eastern students and US educational institutions, it seems that these institutions should educate and inform their faculty and staff about the needs and factors that can ease the process of adjustment. Moreover, to assist Middle Eastern students is to improve their understanding for American customs and culture, and develop good friendships.

Although the current study provides insights on some psychosocial factors affecting the level of adjustment, it might be more judicious to replicate the study broadly using a larger sample size. To maintain the success and retention of Middle Eastern students, faculty and administrators in the US universities should consider the importance of student’s level of adjustment which highly impacts their academic and social performance.

## Data Availability Statement

The original contributions presented in the study are included in the article/supplementary material, further inquiries can be directed to the corresponding author/s.

## Ethics Statement

The studies involving human participants were reviewed and approved by the Committee of Research Ethics and Institutional Review Board at Qassim University. Written informed consent to participate in this study was provided by the participants.

## Author Contributions

The author confirms being the sole contributor of this work and has approved it for publication.
