# Corrigendum: Fighting pressures: successful psychosocial adjustment of middle eastern students at US universities

**DOI:** 10.3389/fpsyg.2024.1365758

**Published:** 2024-01-19

**Authors:** Waleed B. Al Abiky

**Affiliations:** Department of Curriculum and Instruction, College of Education, Qassim University, Buraidah, Saudi Arabia

**Keywords:** successful adjustment, psychosocial, Middle Eastern students, US universities, pressure

In the published article, the Funding statement was omitted in error. The correct Funding statement appears below.

